# Suspected Mpox Symptoms and Testing in Men Who Have Sex With Men in the United States: Cross-Sectional Study

**DOI:** 10.2196/57399

**Published:** 2025-01-16

**Authors:** Kaitlyn Atkins, Thomas Carpino, Amrita Rao, Travis Sanchez, O Winslow Edwards, Marissa Hannah, Patrick S Sullivan, Yasmin P Ogale, Winston E Abara, Kevin P Delaney, Stefan D Baral

**Affiliations:** 1 Department of International Health, Johns Hopkins Bloomberg School of Public Health, Baltimore, MD, United States; 2 Department of Epidemiology, Johns Hopkins Bloomberg School of Public Health, Baltimore, MD, United States; 3 Department of Epidemiology, Emory University Rollins School of Public Health, Atlanta, GA, United States; 4 Centers for Disease Control and Prevention, Atlanta, GA, United States

**Keywords:** mpox, emerging infectious diseases, men who have sex with men, sexual health, testing, monkeypox, epidemic, MSM, United States, rural, cross-sectional study, gay, bisexual, symptomology, sociodemographic, online sample, self-efficacy, rash, fever, HIV, HIV prevention, GBMSM, Black and Hispanic, LGBTQ, Latino, public health, surveillance, sores

## Abstract

**Background:**

The 2022 mpox outbreak in the United States disproportionately affected gay, bisexual, and other men who have sex with men (GBMSM). Uptake of mpox testing may be related to symptomology, sociodemographic characteristics, and behavioral characteristics.

**Objective:**

This study aimed to describe suspected mpox symptoms and testing uptake among a sample of GBMSM recruited via the internet in the United States in August 2022.

**Methods:**

We conducted a rapid internet-based mpox survey from August 5 to 15, 2022, among cisgender men 15 years and older who had previously participated in the 2021 American Men’s Internet Survey. We estimated the prevalence of suspected mpox symptoms (fever or rash or sores with unknown cause in the last 3 mo) and uptake of mpox testing. We calculated adjusted prevalence ratios (aPRs) and 95% CIs for associations between participant characteristics and suspected mpox symptoms and summarized characteristics of GBMSM reporting mpox testing. Among symptomatic GBMSM who did not receive mpox testing, we described testing self-efficacy, barriers, and facilitators.

**Results:**

Of 824 GBMSM, 126 (15.3%) reported at least 1 mpox symptom in the last 3 months; 58/126 (46%) with rash or sores, 57 (45.2%) with fever, and 11 (8.7%) with both. Increased prevalence of suspected mpox symptoms was associated with condomless anal sex (CAS; aPR 1.53, 95% CI 1.06‐2.20). Mpox testing was reported by 9/824 GBMSM (1%), including 5 with symptoms. Most GBMSM reporting mpox testing were non-Hispanic White men (7/9 vs 1 Black and 1 Hispanic or Latino man), and all 9 lived in urban areas. Most reported having an sexually transmitted infections test (8/9), 2 or more partners (8/9), CAS (7/9), and group sex (6/9) in the last 3 months. Of those tested, 3 reported living with HIV and all were on treatment, whereas the remaining 6 men without HIV reported current pre-exposure prophylaxis (PrEP) use. Of symptomatic GBMSM who did not report mpox testing, 47/105 (44.8%) had low mpox testing self-efficacy. Among those with low self-efficacy, the most common barriers to testing were not knowing where to get tested (40/47, 85.1%) and difficulty getting appointments (23/47, 48.9%). Among those with high testing self-efficacy (58/105, 55.2%), the most common facilitators to testing were knowing where to test (52/58, 89.7%), convenient site hours (40/58, 69%), and low-cost testing (38/58, 65.5%).

**Conclusions:**

While all GBMSM who reported testing for mpox were linked to HIV treatment or PrEP, those with symptoms but no mpox testing reported fewer such links. This suggests targeted outreach is needed to reduce structural barriers to mpox services among GBMSM in rural areas, Black and Hispanic or Latino GBMSM, and GBMSM living with HIV. Sustaining and scaling community-tailored messaging to promote testing and vaccination represent critical interventions for mpox control among GBMSM in the United States.

## Introduction

Human mpox is an orthopox virus first identified in the Democratic Republic of the Congo and endemic to regions of Central and Western Africa, which has caused localized outbreaks for decades [1]. mpox is classified into 2 clades; of the two, clade I mpox is associated with more severe symptoms and is highly lethal, with an estimated 10% mortality rate and several thousands of cases annually in Central Africa, including a 2023‐2024 outbreak in the Democratic Republic of the Congo [2]. While clade I mpox has not been observed outside the African continent, clade II mpox has been occasionally reported internationally, often linked to travel [3]. Although clade II infections are associated with a much lower mortality rate, they have resulted in increasing global morbidity, including a global outbreak in 2022 [3]. From 2022 to 2024, there have been 95,000 confirmed cases of clade II mpox globally, including several deaths, predominantly affecting gay, bisexual, and other men who have sex with men (GBMSM) [1].

The first case in the 2022 mpox outbreak was identified in the United States on May 17, 2022, triggering a nationwide response to identify and monitor new cases and deliver the mpox vaccine [4]. As of March 5, 2024, approximately 32,000 mpox cases have been reported in the United States [1], and over 90% of cases in the United States have occurred among cisgender men, the majority of whom had recent sexual contact with a man [5].

As elsewhere, the 2022US mpox outbreak and its disproportionate impacts on GBMSM have been attributed to connected sexual networks. Moreover, the US mpox outbreak demonstrated disparities in infections associated with structural determinants of health such as racism: communities of color reported higher burdens of infections, but lower uptake of testing and vaccines [1,6]. In studies of testing for sexually transmitted infections (STIs) and COVID-19, lower socioeconomic status has been associated with reduced testing uptake [7,8]. However, few studies to date have examined how structural determinants may have shaped mpox testing in a large, national sample of MSM [9,10].

Beyond structural factors, engagement in mpox screening and testing services may be driven by individual factors, particularly risk perception and behavioral indication for mpox tests [11]. For example, GBMSM who report regular condom use have been found to have lower engagement in testing for HIV [12] and bacterial STIs [13]. Condom use and engagement in other sexual behaviors associated with mpox (such as group sex) [14,15] may therefore be associated with increased engagement in mpox testing.

New infections have slowed significantly since the peak of the outbreak [1,16], but equitable testing remains key to contract tracing, treatment, and linkage to vaccination programs. Understanding potential barriers and facilitators to mpox testing during the peak of the 2022 outbreak can inform strategies for future mpox or other STI outbreaks. The current work aims to fill knowledge gaps around these barriers and facilitators by describing suspected mpox symptoms and testing from an mpox study among a sample of prior participants in the American Men’s Internet Survey (AMIS).

## Methods

### Recruitment

The AMIS mpox study was an internet-based survey conducted from August 5 to 15, 2022 in GBMSM aged 15 years and older who had participated in AMIS 2021. Methods for the annual AMIS have been described elsewhere [17]. Annual AMIS participants were recruited via social media and deemed eligible if they identified as cisgender male, aged ≥15 years, had ever had sex with a man, and lived in the United States. For the mpox study, the sampling frame included GBMSM who had completed the AMIS 2021 survey cycle, had sex with a man in the past year, and had consented to be recontacted for future research (N=2999).

### Ethical Considerations

This study received ethical approval from the Emory University Institutional Review Board (IRB00047676). All activities were conducted consistent with US Centers for Disease Control and Prevention policies and regulations. All participants provided informed consent to participate and were not compensated. All study data were deidentified.

### Procedures

Potential participants (ie, all AMIS 2021 participants who had consented to be recontacted for future research, N=2999) were contacted via email with an invitation to participate and a secure survey link. Potential participants were contacted up to twice and provided electronic informed consent after eligibility screening. The internet-based survey included questions in English about demographics, sexual behavior, substance use, HIV and STI testing and diagnosis, HIV pre-exposure prophylaxis (PrEP) use, and mpox knowledge, symptoms, testing, diagnosis, vaccination, stigma, and behavior change.

### Measures

We measured participant age, race or ethnicity (non-Hispanic White, non-Hispanic Black, Hispanic or Latino, other), US census region (Northeast, Midwest, South, or West), urbanicity based on 2013 National Center for Health Statistics classification [18], health insurance (none, private, public, other), self-reported HIV status, current antiretroviral therapy use (among people living with HIV, [19]) and current PrEP use (among those without HIV).

Participants reported, in the past 3 months, their number of sexual partners (1 or ≥2), participation in group sex, bathhouses or sex clubs, or sex parties, and any illicit or injection drug use. They also reported condomless anal sex (CAS) in the past 12 months, CAS with partners of a different HIV status in the past 3 months, HIV testing in the past 12 months, and STI testing in the past 3 months.

#### Outcomes

We assessed symptoms suggesting mpox by asking: “In the past three months, have you had a (1: fever or 2: new rash/sores on the skin) and didn’t know the cause?” For GBMSM reporting rashes, we asked their location and pain severity (rated 0‐10), and whether, after GBMSM first noticed the rash or sores, they changed in appearance, number, or location.

We measured mpox testing by self-report. For GBMSM reporting no mpox testing, we assessed agreement (4-point Likert scale) with the statement: “If I thought I had mpox, I could get a test if I wanted it.” GBMSM who strongly or somewhat agreed were considered to have high mpox testing self-efficacy, and those who strongly or somewhat disagreed as having low self-efficacy. We also asked participants what made it easy (for those with high self-efficacy) or difficult (for those with low self-efficacy) to get a test; parallel response options included location, eligibility, appointments, using one’s own provider, site times, privacy, and cost.

### Statistical Analysis

We calculated frequencies for participant characteristics, symptoms suggesting mpox, and mpox testing. We calculated unadjusted prevalence ratios (PRs) and 95% CIs for associations between participant characteristics and symptoms suggesting mpox. Factors found to have a significant association (α<.05) with symptoms were included in a multivariable model to calculate adjusted prevalence ratios (aPRs). The final model included age, race or ethnicity, PrEP use, STI testing, and CAS. We assessed model fit using Hosmer-Lemeshow test, which has been recommended for use with binomially distributed data [20]. We used frequencies to summarize the characteristics of GBMSM reporting mpox testing. Among symptomatic GBMSM who did not report mpox testing, we summarized testing self-efficacy, barriers, and facilitators. All analyses were performed in Stata 15.1 (StataCorp) [21].

## Results

### Sample Characteristics

Of 2999 GBMSM in the sampling frame, 824 completed the mpox study survey for a response rate of 27.5%. The majority of mpox study participants were aged ≥40 years (478/824, 58%), non-Hispanic White (581/824, 70.9%), residents of urban areas (770/824, 93.4%), and had private health insurance (564/824, 68.6%, Table 1). In the last 3 months, a minority reported CAS with a partner of a different HIV status (220/824, 26.7%), group sex (238/824, 29.1%), or attending a sex club (147/824, 19.7%), or sex party (111/824, 14.9%). Just over two-thirds (575/824, 69.8%) reported past-year HIV testing, and around half (408/824, 49.6%) reported STI testing in the last 3 months. Most (720/824, 87.4%) were not people living with HIV and 44.7% of these (316/720, 44.7%) were currently using PrEP; of 104 people living with HIV in the sample, 99 (96.1%) were currently on HIV treatment.

**Table 1.** Characteristics of participants in the American Men’s Internet Survey mpox substudy, United States, August 2022 (n=824).

Characteristics		Participants (n=824), n (%)
**Age (years)**		
	15‐24	46 (5.6)
	25‐29	86 (10.4)
	30‐39	214 (26)
	40 or older	478 (58)
**Race or ethnicity**		
	Non-Hispanic White	581 (70.9)
	Non-Hispanic Black	87 (10.6)
	Hispanic or Latino	88 (10.7)
	Other	64 (7.8)
**US census region**		
	Northeast	174 (21.1)
	Midwest	141 (17.1)
	South	305 (37)
	West	204 (24.8)
**Urbanicity**		
	Rural	54 (6.6)
	Urban	770 (93.4)
**Health insurance**		
	None	24 (2.9)
	Private	564 (68.6)
	Public	164 (20)
	Other or multiple	70 (8.5)
**HIV test, last 12 months**		
	No	249 (30.2)
	Yes	575 (69.8)
**Bacterial STI^a^ test, last 3 months**		
	No	410 (49.8)
	Yes	409 (49.6)
**HIV status**		
	Not living with HIV	720 (87.4)
	Living with HIV	104 (12.6)
**Current ART^b^ use (n=103)^c^**		
	No	4 (3.9)
	Yes	99 (96.1)
**Current PrEP^d^ use (** **n=707)**		
	No	391 (55.3)
	Yes	316 (44.7)
**Group sex, last 3 months**		
	No	580 (70.9)
	Yes	238 (29.1)
**Sex club, last 3 months**		
	No	599 (80.3)
	Yes	147 (19.7)
**Sex party, last 3 months**		
	No	634 (85.1)
	Yes	111 (14.9)
**CAS^e^ with serodifferent partner, last 3 months**		
	No	604 (73.3)
	Yes	220 (26.7)

a STI: sexually transmitted infection.

b ART: antiretroviral therapy.

c One participant with HIV did not provide information on ART use.

d PrEP: HIV pre-exposure prophylaxis.

e CAS: condomless anal sex.

### Prevalence of Symptoms Suggesting mpox and Associated Factors

In total, 126/824 (15.3%) participants reported at least 1 mpox symptom in the last 3 months (Table 2): 58/126 (46%) with rash or sores, 57 (45%) with fever, and 11 (9%) with both. In unadjusted analyses, there was decreased prevalence of symptoms suggesting mpox among participants aged ≥40 years versus 15‐24 years (PR 0.48, 95% CI 0.28‐0.82) and among Hispanic or Latino versus non-Hispanic White participants (PR 0.44, 95% CI 0.20‐0.97), and increased prevalence among participants reporting current PrEP use (PR 1.48, 95% CI 1.05‐2.09), recent STI tests (PR 1.40, 95% CI 1.01‐1.95), and CAS in the last 12 months (PR 1.64, 95% CI 1.14‐2.35). In our adjusted model, we failed to reject the Hosmer-Lemeshow null hypothesis (Hosmer-Lemeshow *χ*_8_^2^5.01; *P*=.76), indicating adequate model fit [20]. In adjusted analyses, prevalence of symptoms suggesting mpox remained lower among GBMSM aged ≥40 years versus 15‐24 years (aPR 0.49, 95% CI 0.28‐0.83), and higher among those reporting CAS (aPR 1.53, 95% CI 1.06‐2.20).

**Table 2.** Symptoms suggesting mpox and testing among participants in the American Men’s Internet Survey mpox substudy, United States, August 2022 (n=824). Bold text indicates measures of association found to be statistically significant at α<.05; that is, confidence intervals that did not cross 1.00.

		At least 1 symptom suggesting mpox^a^, last 3 months			Symptoms, no mpox testing	Ever tested for mpox	
		Participants (n=126), n (%)	PR^b^ (95% CI)	aPR^c^ (95% CI)	Participants (n=114), n (%)	Participants (n=9), n (%)	
**Symptoms suggesting mpox**							
	No fever or rash	—^d^	—	—	—	4 (44.4)	
	Fever only	57 (45.2)	—	—	53 (46.5)	0 (0)	
	Rash or sores only	58 (46)	—	—	52 (45.6)	4 (44.4)	
	Fever and rash or sores	11 (8.7)	—	—	9 (7.9)	1 (11.1)	
**Age (years)**							
	15‐24	12 (9.5)	REF^e^	REF	10 (8.8)	1 (11.1)	
	25‐29	14 (11.1)	0.61 (0.31‐1.21)	0.64 (0.33‐1.26)	12 (10.5)	2 (22.2)	
	30‐39	39 (31)	0.68 (0.39‐1.20)	0.64 (0.37‐1.12)	36 (31.6)	3 (33.3)	
	40 or older	61 (48.4)	0.48 (0.28‐0.82)	0.49 (0.28‐0.83)	56 (49.1)	3 (33.3)	
**Race or ethnicity**							
	Non-Hispanic White	90 (71.4)	REF	REF	81 (71.1)	7 (77.8)	
	Non-Hispanic Black	14 (11.1)	1.03 (0.62‐1.73)	1.01 (0.60‐1.71)	12 (10.5)	1 (11.1)	
	Hispanic or Latino	6 (4.8)	0.44 (0.20‐0.97)	0.41 (0.19‐0.91)	5 (4.4)	1 (11.1)	
	Other	16 (12.7)	1.63 (1.02‐2.59)	1.44 (0.90‐2.30)	16 (14)	0 (0)	
**Urbanicity**							
	Nonurban	11 (8.7)	REF	—	10 (8.8)	0 (0)	
	Urban	115 (91.3)	0.74 (0.42‐1.28)	—	104 (91.2)	9 (100)	
**HIV status**							
	Not living with HIV	111 (88.1)	REF	—	102 (89.5)	6 (66.7)	
	Living with HIV	15 (11.9)	0.93 (0.56‐1.53)	—	12 (10.5)	3 (33.3)	
**Current ART^f^ use (n=15)**							
	No	0 (0)	—	—	0 (0)	0 (0)	
	Yes	15 (100)	—	—	12 (100)	3 (100)	
**Current PrEP^g^ use (n=707)^h^** ^,^ ^**i**^							
	No	50 (45.5)	REF	REF	47 (46.5)	0 (0)	
	Yes	60 (54.5)	1.48 (1.05‐2.09)	1.15 (0.75‐1.75)	54 (53.5)	6 (100)	
**STI^j^ test, last 3 months**							
	No	52 (41.3)	REF	REF	45 (39.5)	1 (11.1)	
	Yes	74 (58.7)	1.40 (1.01‐1.95)	1.33 (0.96‐1.85)	69 (60.5)	8 (88.9)	
**Number of partners, last 3 months**							
	One	25 (21)	REF	—	23 (21.3)	0 (0)	
	Two or more	94 (79%)	1.44 (0.95‐2.17)	—	85 (78.7)	8 (100)	
**Group sex, last 3 months**							
	No	81 (64.3%)	REF	—	76 (66.7)	3 (33.3)	
	Yes	45 (35.7)	1.35 (0.97‐1.88)	—	38 (33.3)	6 (66.7)	
**Sex club, last 3 months**							
	No	88 (74)	REF	—	81 (75)	5 (62.5)	
	Yes	31 (26)	1.44 (1.00‐2.08)	—	27 (25)	3 (37.5)	
**Sex party, last 3 months**							
	No	96 (80.7)	REF	—	89 (82.4)	5 (62.5)	
	Yes	23 (19.3)	1.36 (0.91‐2.05)	—	19 (17.6)	3 (37.5)	
**CAS^k^**, **last 12 months**							
	No	36 (28.6)	REF	REF	33 (28.9)	2 (22.2)	
	Yes	90 (71.4)	1.64 (1.14‐2.35)	1.53 (1.06‐2.20)	81 (71.1)	7 (77.8)	

a mpox: mpox.

b PR: prevalence ratio.

c aPR: adjusted prevalence ratio.

d Not applicable.

e REF: reference.

f ART: antiretroviral therapy.

g PrEP: HIV pre-exposure prophylaxis.

h The model with PrEP use was restricted to individuals without HIV, and included all listed covariates except HIV status.

i No PR reported given 100% of symptomatic people living with HIV reported current antiretroviral therapy use.

j STI: sexually transmitted infection.

k CAS: condomless anal sex.

### Uptake of mpox Testing

Among 126 symptomatic GBMSM, 114 (90.5%) did not report mpox testing; their characteristics mirrored those of the overall sample (Table 2). mpox testing was reported by 9 participants (1%, Table 2), including 5 with reported symptoms. Most GBMSM reporting mpox testing were non-Hispanic White (7/9 vs 1 Black and 1 Hispanic or Latino man) and all 9 lived in urban areas. Most reported STI testing (8/9), 2 or more partners (8/9), CAS (7/9), and group sex (6/9) in the last 3 months. Three were people living with HIV, all on treatment; the remaining 6 without HIV reported current PrEP use.

Among 69 GBMSM experiencing recent rashes or sores with unknown cause, most located them on hands or arms, feet or legs, and genitals or pelvic area (Table 3). Over half (31/50, 62%) rated their pain as ≤2 on a 10-point scale. Most (38/69, 55%) reported no changes over time; the most common reported change was worsening appearance of rash or sores (22/69, 32%).

**Table 3.** Rash or sore location, severity, and changes among men having sex with men in the United States reporting new rashes or sores in the last 3 months (n=69).

		Experienced new rash or sores with unknown cause, last 3 months (n=69), n (%)
**Location of rash**		
	Hands or arms	26 (37.7)
	Feet or legs	24 (34.8)
	Genitals or pelvic area	19 (27.5)
	Chest	14 (20.3)
	Face	13 (18.8)
	Anus	10 (14.5)
	Mouth	7 (10.1)
**Severity of rash or sores**		
	0: no pain at all	10 (20)
	1‐2	21 (42)
	3‐4	11 (22)
	5‐6	4 (8)
	7‐8	4 (8)
	9‐10: the worst pain possible	0 (0)
**Changes in rash or sores**		
	No changes	38 (55.1)
	Worsening appearance	22 (31.9)
	Increased number of rashes or sores in the same location	11 (15.9)
	Rashes or sores in new locations	7 (10.1)

Of the 126 symptomatic GBMSM, 105 (83%) did not report mpox testing and were offered questions about testing self-efficacy and barriers and facilitators to testing (Table 4). Among GBMSM with high testing self-efficacy (58/105, 55%), common facilitators to testing were knowing where to test (90%), convenient site hours (69%), and low-cost testing (38/58, 66%). Among those with low testing self-efficacy (47/105, 45%), the most common barriers to testing were not knowing where to get tested (40/47, 85%) and difficulty getting appointments (23/47, 49%).

**Table 4.** Characteristics of gay, bisexual, and other men who have sex with men with symptoms suggesting mpox who did not take up mpox testing (n=105).

Barriers or facilitators to testing		Low testing self-efficacy^a^ (n=47), n (%)	High testing self-efficacy (n=58), n (%)
(Not) knowing where to get tested		40 (85.1)	52 (89.7)
Getting an appointment		23 (48.9)	29 (50)
Getting tested at own doctor		17 (36.2)	24 (41.4)
Convenience or testing site hours		11 (23.4)	40 (69)
(Not) knowing who is eligible		10 (21.3)	29 (50)
Cost considerations		7 (14.9)	38 (65.5)
Privacy considerations		6 (12.8)	19 (32.8)

a Assessed using the statement: “If I thought I had mpox, I could get a test if I wanted it.” Participants who strongly or somewhat agreed were considered to have high mpox testing self-efficacy, and those who strongly or somewhat disagreed as having low self-efficacy.

## Discussion

### Principal Findings

Our findings show that a large proportion of GBMSM with symptoms suggesting mpox did not access mpox testing and mpox testing was low or zero among GBMSM in rural areas and GBMSM who were younger, Black, Hispanic or Latino, or people living with HIV.

Compared with non-Hispanic White participants, we observed low levels of mpox testing among Black and Hispanic or Latino GBMSM in this study. While this likely reflects our overall sample, which was primarily non-Hispanic White, it is worth noting that this finding aligns with broader disparities not only in mpox cases and vaccine delivery, but also with the majority of US vaccine doses administered to date to non-Hispanic White individuals [1]. Our findings may reflect structural challenges with communication and knowledge of access to and locations of testing, laboratory capacity, and stigma [22]. As others have noted, additional data are needed to ensure equitable access to and communication on mpox testing, including routine collection of demographic data through testing programs [23,24].

There was no reported testing among GBMSM in rural or nonurban areas in our study. While few participants in our study were from rural areas, this parallels findings from an internet-based study also conducted in August 2022, which found that GBMSM in rural areas had lower perceived susceptibility to and severity of mpox, compared with urban GBMSM [25]. The authors of the earlier manuscript found further disparities in rural GBMSM’s perceptions of mpox vaccine benefits and in their intention to be vaccinated, amidst potentially inadequate vaccine supply. Our findings likely reflect broader challenges with public health infrastructure in rural areas [26] and increased availability of HIV and STI services in urban areas, which may have facilitated linkages to mpox testing. While there are opportunities to leverage these services for mpox testing, given only half of potentially symptomatic GBMSM were engaged with PrEP and STI services, additional outreach may be needed.

Testing uptake was also low among GBMSM living with HIV. While only about 1 in 5 participants in our study were people living with HIV, we agree with others that tailored interventions can better serve this community with services, including mpox testing, postexposure prophylaxis for mpox exposure, and vaccination [27]. Current US surveillance data suggest that around 40% of mpox diagnoses were among people living with HIV [1]. Given recent evidence that suggests severe mpox in the context of advanced immunosuppression may be an AIDS-defining condition [28], structural interventions to improve access to mpox testing among people living with HIV, such as integrating mpox testing into routine care for people living with HIV, are critical to ensure early identification and treatment of new mpox cases.

We found that the most symptoms suggesting mpox in our sample were rashes or sores, most commonly in the arms, legs, and genitals. Rashes in the arms and legs were reported at a similar rate in our study as has been shown in broader US surveillance data; however, we saw less frequent rashes in the genitals and face [5]. In our study, rashes and sores were reported to be generally mild and commonly did not change after they were first observed. This may explain the relatively low uptake of mpox testing among potentially symptomatic GBMSM, because GBMSM with mild symptoms suggesting mpox may not have felt they needed a test or been eligible for testing at that time [5]. During clinical encounters with GBMSM (eg, STI testing), providers should continue to counsel GBMSM to seek mpox testing and vaccine if they develop unexplained rash or other symptoms consistent with mpox.

### Limitations

A key limitation of these analyses is the small sample of GBMSM in our study who reported mpox testing (n=9), precluding advanced statistical analyses comparing the characteristics of GBMSM who tested for mpox with those who did not. Furthermore, we are unable to draw generalizable conclusions based on the characteristics of these 9 individuals. We recruited a convenience sample that is subject to selection bias; our respondents were predominantly non-Hispanic White, elderly, and privately insured and may have increased access to mpox testing services—the sample is thus unlikely to represent all GBMSM in the United States. Our use of self-reported data and recall period of 3 months for most outcomes may have introduced recall bias; furthermore, we are unable to confirm whether those with symptoms suggesting mpox indeed had acquired mpox. Given symptoms were reported in the last 3 months, in some cases, participant responses may have reflected symptoms before the first US mpox case on May 17, 2022. We were also not able to place individuals’ locations at the time of symptom onset or testing, which may have affected testing uptake given the nonuniform roll-out of testing nationwide. Regarding symptoms, we only included items assessing the presence of fever and rash or sores; we did not inquire about other symptoms commonly reported among mpox cases such as malaise, headache, nausea or vomiting, pain, itching, or bleeding. Low mpox testing prevalence in this sample may be attributed to perceptions that symptoms, such as fever or rash, were unrelated to mpox and therefore did not require testing. Our findings also reflect the timing of data collection (August 2022); access to mpox testing and other services likely changed as the US outbreak response evolved. For example, testing uptake could have been limited due to sustained increases of telehealth services after the COVID-19 pandemic, particularly among wealthier, insured patients such as the GBMSM who comprised the majority of our sample [29]. Furthermore, access to testing over the course of the outbreak likely varied by geographic location, something we were unable to fully assess in this analysis.

### Conclusions

Similar structural barriers that have long been known to increase the risk of other STIs were rapidly replicated during the mpox outbreak in 2022. Sustained disparities fostered by racism, stigma, and limited access to health care in rural settings contribute to ongoing morbidity and mortality for GBMSM and predispose many communities to unexpected new outbreaks and epidemics such as mpox. While the epidemiology of mpox continues to evolve, transmission risk remains present and characterizing optimal public health interventions for mpox is critical to both prevent future outbreaks and more rapidly respond if they occur.

## Abbreviations

AMIS: American Men’s Internet Survey
aPR: adjusted prevalence ratio
CAS: condomless anal sex
GBMSM: gay, bisexual, and other men who have sex with men
PR: prevalence ratio
PrEP: pre-exposure prophylaxis
STI: sexually transmitted infection
